# Cross-sectional and longitudinal association of sleep and Alzheimer biomarkers in cognitively unimpaired adults

**DOI:** 10.1093/braincomms/fcac257

**Published:** 2022-11-03

**Authors:** Jonathan Blackman, Laura Stankeviciute, Eider M Arenaza-Urquijo, Marc Suárez-Calvet, Gonzalo Sánchez-Benavides, Natalia Vilor-Tejedor, Alejandro Iranzo, José Luis Molinuevo, Juan Domingo Gispert, Elizabeth Coulthard, Oriol Grau-Rivera, for the European Prevention of Alzheimer’s Disease (EPAD) Consortium

**Affiliations:** North Bristol NHS Trust, Bristol BS10 5NB, UK; Bristol Medical School, University of Bristol, Bristol BS8 1UD, UK; Barcelonaβeta Brain Research Center (BBRC), Pasqual Maragall Foundation, Barcelona 08005, Spain; Universitat Pompeu Fabra, Barcelona 08005, Spain; Barcelonaβeta Brain Research Center (BBRC), Pasqual Maragall Foundation, Barcelona 08005, Spain; IMIM (Hospital del Mar Medical Research Institute), Barcelona 08003, Spain; Centro de Investigación Biomédica en Red de Fragilidad y Envejecimiento Saludable (CIBERFES), Madrid 28029, Spain; Barcelonaβeta Brain Research Center (BBRC), Pasqual Maragall Foundation, Barcelona 08005, Spain; IMIM (Hospital del Mar Medical Research Institute), Barcelona 08003, Spain; Centro de Investigación Biomédica en Red de Fragilidad y Envejecimiento Saludable (CIBERFES), Madrid 28029, Spain; Servei de Neurologia, Hospital del Mar, Barcelona 08003, Spain; Barcelonaβeta Brain Research Center (BBRC), Pasqual Maragall Foundation, Barcelona 08005, Spain; IMIM (Hospital del Mar Medical Research Institute), Barcelona 08003, Spain; Centro de Investigación Biomédica en Red de Fragilidad y Envejecimiento Saludable (CIBERFES), Madrid 28029, Spain; Barcelonaβeta Brain Research Center (BBRC), Pasqual Maragall Foundation, Barcelona 08005, Spain; Universitat Pompeu Fabra, Barcelona 08005, Spain; Centre for Genomic Regulation (CRG), The Barcelona Institute for Science and Technology, Barcelona 08003, Spain; Department of Clinical Genetics, Erasmus University Medical Center, Rotterdam 3015 GD, The Netherlands; Neurology Service, Hospital Clínic de Barcelona and Institut D'Investigacions Biomèdiques, Barcelona 08036, Spain; Centro de Investigación Biomédica en Red sobre Enfermedades Neurodegenerativas (CIBERNED), Hospital Clínic de Barcelona, Barcelona 28029, Spain; Barcelonaβeta Brain Research Center (BBRC), Pasqual Maragall Foundation, Barcelona 08005, Spain; Barcelonaβeta Brain Research Center (BBRC), Pasqual Maragall Foundation, Barcelona 08005, Spain; IMIM (Hospital del Mar Medical Research Institute), Barcelona 08003, Spain; Centro de Investigación Biomédica en Red de Bioingeniería, Biomateriales y Nanomedicina (CIBER-BBN), Madrid 28029, Spain; North Bristol NHS Trust, Bristol BS10 5NB, UK; Bristol Medical School, University of Bristol, Bristol BS8 1UD, UK; Barcelonaβeta Brain Research Center (BBRC), Pasqual Maragall Foundation, Barcelona 08005, Spain; IMIM (Hospital del Mar Medical Research Institute), Barcelona 08003, Spain; Centro de Investigación Biomédica en Red de Fragilidad y Envejecimiento Saludable (CIBERFES), Madrid 28029, Spain; Servei de Neurologia, Hospital del Mar, Barcelona 08003, Spain

**Keywords:** Alzheimer’s disease, biomarkers, sleep, preclinical

## Abstract

Sleep abnormalities are prevalent in Alzheimer’s disease, with sleep quality already impaired at its preclinical stage. Epidemiological and experimental data point to sleep abnormalities contributing to the risk of Alzheimer’s disease. However, previous studies are limited by either a lack of Alzheimer’s disease biomarkers, reduced sample size or cross-sectional design. Understanding if, when, and how poor sleep contributes to Alzheimer’s disease progression is important so that therapies can be targeted to the right phase of the disease. Using the largest cohort to date, the European Prevention of Alzheimer’s Dementia Longitudinal Cohort Study, we test the hypotheses that poor sleep is associated with core Alzheimer’s disease CSF biomarkers cross-sectionally and predicts future increments of Alzheimer’s disease pathology in people without identifiable symptoms of Alzheimer’s disease at baseline. This study included 1168 adults aged over 50 years with CSF core Alzheimer’s disease biomarkers (total tau, phosphorylated tau and amyloid-beta), cognitive performance, and sleep quality (Pittsburgh sleep quality index questionnaire) data. We used multivariate linear regressions to analyse associations between core Alzheimer’s disease biomarkers and the following Pittsburgh sleep quality index measures: total score of sleep quality, binarized score (poor sleep categorized as Pittsburgh sleep quality index > 5), sleep latency, duration, efficiency and disturbance. On a subsample of 332 participants with CSF taken at baseline and after an average period of 1.5 years, we assessed the effect of baseline sleep quality on change in Alzheimer’s disease biomarkers over time. Cross-sectional analyses revealed that poor sleep quality (Pittsburgh sleep quality index total > 5) was significantly associated with higher CSF t-tau; shorter sleep duration (<7 h) was associated with higher CSF p-tau and t-tau; and a higher degree of sleep disturbance (1–9 versus 0 and >9 versus 0) was associated with lower CSF amyloid-beta. Longitudinal analyses showed that greater sleep disturbances (1–9 versus 0 and >9 versus 0) were associated with a decrease in CSF Aβ42 over time. This study demonstrates that self-reported poor sleep quality is associated with greater Alzheimer’s disease-related pathology in cognitively unimpaired individuals, with longitudinal results further strengthening the hypothesis that disrupted sleep may represent a risk factor for Alzheimer’s disease. This highlights the need for future work to test the efficacy of preventive practices, designed to improve sleep at pre-symptomatic stages of disease, on reducing Alzheimer’s disease pathology.

## Teaser

This study tested associations between self-reported sleep abnormalities and CSF biomarkers of Alzheimer’s Disease in a large cohort of cognitively unimpaired individuals. Cross-sectionally, higher CSF tau was associated with overall sleep quality/duration with CSF amyloid-beta associated with sleep disturbances. Further, sleep disturbances predicted decreasing CSF amyloid-beta longitudinally.

## Graphical Abstract

**Graphical abstract:**
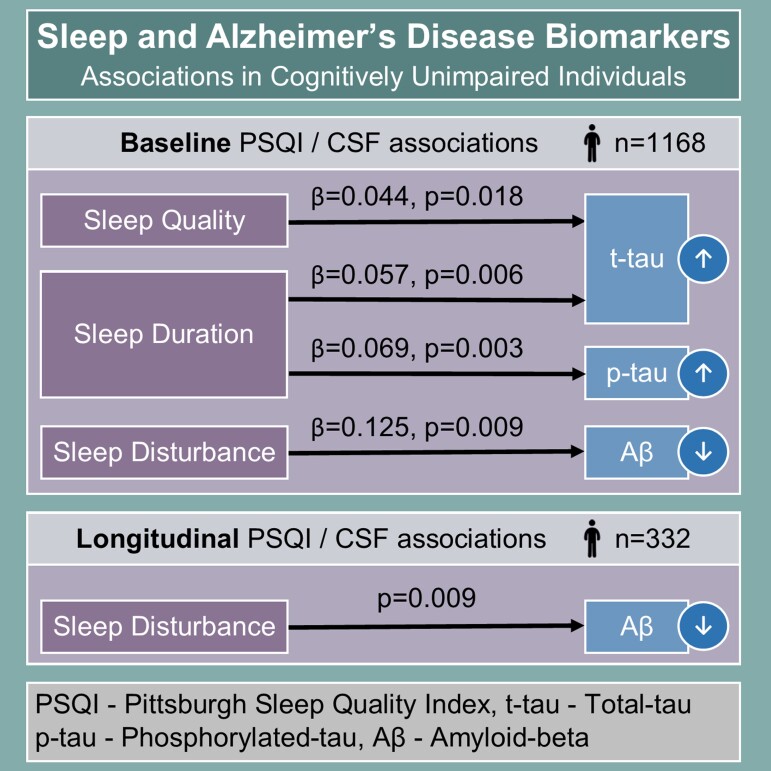

## Introduction

Sleep disturbance and circadian rhythm disorders are well recognized as intrinsic symptoms of established Alzheimer’s Disease.^1–6^ Alzheimer’s disease dementia is associated with a broad range of sleep macro-architectural changes, including reduced total sleep time, excessive daytime sleepiness, decreased sleep efficiency and increased sleep fragmentation,^7^ with the extent of abnormalities correlating with dementia severity.^3,7–10^ Sleep abnormalities are also well described earlier in the natural history of Alzheimer’s disease, during and even preceding the mild cognitive impairment (MCI) stage.^3,11–16^ In addition, a growing body of literature recognizes insomnia and conditions associated with fragmented sleep as independent risk factors for Alzheimer’s disease dementia.^13–15,17^

Abnormalities in sleep may reflect early symptomatic manifestations of Alzheimer’s disease pathology, however, there are also plausible mechanisms by which sleep disturbances could hasten pathophysiology, specifically through the loss of sleep’s modulatory role in governing concentrations of the key metabolites in the pathognomonic changes of Alzheimer’s disease.^18^

CSF biomarkers, including amyloid-β 42 (Aβ42), total tau (t-tau) and phosphorylated tau (p-tau), reflect key aspects of Alzheimer’s disease pathophysiology, correlating well with amyloid PET,^19^ and have been validated in providing early high diagnostic accuracy.^20,21^ Sleep-wake activity has been shown to affect their production, release, clearance (via the glymphatic system) and metabolism.^22,23^ However, the precise nature of sleep abnormalities and even the direction of its correlation with Alzheimer’s disease CSF biomarkers has not been consistently reported in the literature.

Experimental studies have shown that acute sleep deprivation increases interstitial fluid (ISF) and CSF levels of Aβ in humans and animal models.^24–26^ However, cross-sectional observational studies have yielded mixed results. Lower actigraphy-measured sleep efficiency and self-reported increased daytime napping have been associated with lower CSF Aβ42 levels in cognitively unimpaired middle-aged adults.^27^ Similarly, lower CSF Aβ42/Aβ40, higher t-tau/Aβ42 and p-tau/Aβ42 levels have been associated with worse subjective sleep quality and daytime somnolence,^28,29^ as well as both reduced and excessive sleep duration in cognitively unimpaired adults.^29^ Yet, higher levels of CSF Aβ42 have been found to be associated with self-reported insomnia,^30^ and also with reduced slow-wave activity and more fragmented slow-wave sleep in cognitively unimpaired adults.^31^

The reported relationship between CSF tau and sleep disturbance has also been inconsistent. Previous studies have shown that sleep restriction increases CSF and ISF tau levels in mouse models and humans,^32,33^ potentially through compromised glymphatic system activity.^34^ However, others have not reported this association, possibly due to the longer turnover time of tau when compared to Aβ.^25,35–37^ In cross-sectional observational studies, one study found no differences in CSF tau levels when comparing patients with insomnia to controls.^30^ Conversely, poor sleep quality over several days has been associated with increased CSF tau in healthy adults^25^ and a faster rate of CSF tau accumulation has been reported in adults with obstructive sleep apnoea (OSA) compared with controls.^38^

Several reasons may explain these inconsistencies across published findings. First, most studies are cross-sectional, thereby restricting inferences regarding important dynamic effects of sleep on CSF biomarkers over time. Second, few studies have explored sleep quality in preclinical Alzheimer’s disease, despite this intuitively reflecting the optimum stage for intervention, before architectural changes associated with neurodegeneration have become established. Third, there is significant methodological heterogeneity between studies. Specifically, the use of objective versus subjective sleep measures makes comparison difficult, due to the lack of perfect correlation in these measures more apparent at the earliest disease stages.^39^ Last, aside from one larger cross-sectional study of 736 participants,^29^ studies exploring this relationship have been small in sample size.

This study tests the hypotheses that baseline self-reported poor sleep quality in cognitively unimpaired individuals is associated cross-sectionally with a higher burden of Alzheimer’s disease pathology, and with its accumulation over time. These hypotheses are tested in the largest cohort to date using both cross-sectional and longitudinal data to assess the association between subjective sleep quality and Alzheimer’s disease CSF biomarkers. Given the high prevalence associated with both sleep disturbances in the elderly population and MCI/Alzheimer’s disease groups, investigating plausible neurobiological underpinnings of this relationship may enhance understanding of the neurodegenerative processes and clinical trajectory. Disentangling this link may reveal sleep as a target for treatment and prevention strategies. As effective treatments for sleep disturbances exist, they could be rapidly implemented to mitigate cognitive decline when targeted to an appropriate stage of the Alzheimer’s disease continuum.

## Materials and methods

### Participants and study design

This cross-sectional and longitudinal study includes participants from The European Prevention of Alzheimer’s Dementia Longitudinal Cohort Study (EPAD-LCS) registered at www.clinicaltrials.gov identifier NCT02804789. The primary research goal of the EPAD-LCS is to provide a well-phenotyped probability-spectrum population for developing and continuously improving disease models for Alzheimer’s disease in individuals without dementia.

The cohort comprises over 2000 adults aged 50 years or older without a diagnosis of dementia. Key exclusion criteria included severe medical co-morbidity or major neurological disorders (for full criteria see Supplementary Table 1). Research participants were characterized with MRI, CSF Alzheimer’s disease biomarkers, standard cognitive assessment and genetic data. Additionally, information was collected regarding lifestyle factors including sleep habits [Pittsburgh Sleep Quality Index (PSQI)], smoking habits, alcohol consumption, diet and physical activity variables. Full details of participant selection and methods are described within its study protocol.^40,41^

**Figure 1:**
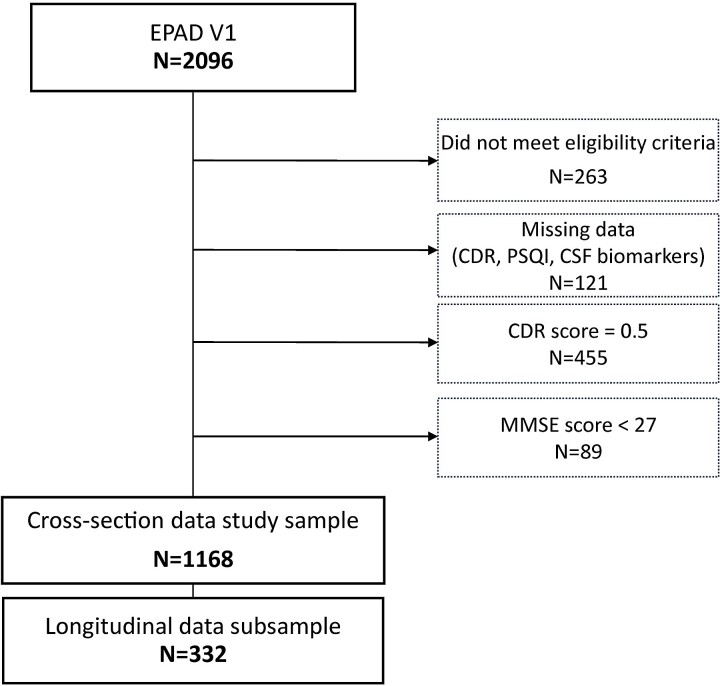
**The flow-chart illustrating stepwise exclusion process of participants used in this study**.

Data used in preparation of this article were obtained from the EPAD-LCS data set V.IMI (doi:10.34688/epadlcs_v.imi_20.10.30) comprising 2096 EPAD participants enrolled from 2016 to 2020 (see Fig. 1).

Participants were excluded as a result of ineligibility for full EPAD participation, *n* = 263; missing data [Clinical Dementia Rating (CDR) score, PSQI or Alzheimer’s disease CSF biomarkers], *n* = 121; CDR score = 0.5, *n* = 455; and MMSE < 27, *n* = 89, leaving a final sample consisting of 1168 cognitively unimpaired individuals (CDR: 0).

We analysed cross-sectional effects of self-reported sleep measures in the whole sample but also investigated longitudinal changes in CSF biomarkers, where this data was available in a subsample of 332 individuals.

### Sleep assessment

The Pittsburgh Sleep Quality Index (PSQI), a brief, 19-item, self-rated questionnaire assessing sleep quality over the preceding month, provides the measure of subjective sleep quality for this study. PSQI scoring is based on seven components that assess different sleep-related domains: (i) subjective sleep quality, (ii) latency of sleep, (iii) length of sleep, (iv) sleep efficiency, (v) sleep disturbances, (vi) use of sleep medicines, and (vii) daytime dysfunction. Each component is scored on a scale from 0 to 3, with 3 indicating the extreme negative evaluation—severe difficulty. Finally, all component scores are summated yielding a global score (0–21). A total score above 5 is indicative of poor quality of sleep.^42^ The PSQI was repeated at each follow-up visit.

### CSF samples

All participants underwent lumbar puncture at baseline and CSF samples were obtained following a harmonised protocol.^41^ In 332 participants, more than one CSF sample was collected during follow-up. Among these, 268 (80.7%) had CSF samples from two separated time points, 63 (19.0%) from three time points and one (0.3%) from four time points. The interval of time between the first and last CSF sample collection was on average 1.5 years (SD 0.5). Total tau (t-tau), p-tau, and Aβ42 levels were measured with fully automatised ElectroChemiLuminescence Roche Elecsys® System immunoassays at the University of Gothenburg from CSF samples obtained using a standard protocol.^43^

### Neuropsychological evaluation

EPAD participants underwent a standardised neuropsychological examination battery that included screening tests such as the Mini-Mental State Examination (MMSE)^44^ and the CDR scale.^45^ The Geriatric Depression Scale (GDS) and State-Trait Anxiety Inventory (STAI) were used for the assessment of psychological status.^46,47^

### Statistical analysis

Outliers were excluded utilising Tukey’s criteria set at three times the interquartile range. Normality was assessed visually and by the Shapiro-Wilk test. Non-parametrically distributed variables CSF Aβ42, p-tau and t-tau levels were log_10_-transformed. For all analyses, a 2-tailed *P* < 0.05 was considered significant.

Multivariate linear regression analyses were used to assess the relationships between sleep variables yielded by the PSQI questionnaire as predictors, and continuous CSF biomarkers (Aβ42, t-tau and p-tau) as outcomes. The following PSQI measures were used: total score of sleep quality, binarized score (poor sleep quality categorised as PSQI >5), sleep latency, sleep efficiency, sleep duration, sleep disturbances, and daytime dysfunction. Reference categories for categorical variables reflected optimum sleep quality/duration or daytime function (PSQI ≤ 5 for binarized PSQI, sleep latency ≤ 15 min, sleep duration > 7 h, sleep efficiency > 85%, sleep disturbances score of 0, and daytime dysfunction score of 0). For each PSQI component, adjacent categories were collapsed whenever the number of observations in any category was less than 20 [*e.g.* baseline sleep disturbances score of 10–18 (*n* = 250) was merged with a score of 19–27 (*n* = 6)]. Separate models defined each biomarker as the dependent variable with each sleep measure as the predictor. All models were adjusted by core covariates—age, sex, research site and *APOE*-ɛ4 status (carriers versus non-carriers). In order to adjust by additional potential confounders but minimise data overfitting, we adjusted by additional confounders only if found to be significant (*P*-value (*P* < 0.05) in a saturated model. Potential confounders assessed in this model included depression (GDS), anxiety (STAI), physical activity, body mass index (BMI) and sleep medication (dichotomized PSQI component 6 variable—use of sleep medication less than once a week versus at least once a week). To see if the effect of sleep measures on each biomarker was independent from other biomarkers, we further adjusted all models by other biomarkers’ baseline levels. Following this procedure, models with CSF Aβ42 were adjusted by core covariates, anxiety (STAI) and log_10_(CSF p-tau), and models with CSF t-tau or p-tau as outcomes, were adjusted by core covariates, physical activity, BMI and log_10_(CSF Aβ42).

For cross-sectional analyses we also performed binary logistic regression models where our outcome measures were dichotomic variables of CSF biomarkers based on established cut-offs: Aβ-positive: CSF Aβ42 < 1000 pg/ml, p-tau-positive: CSF p-tau > 27 pg/ml, t-tau-positive: CSF p-tau > 300 pg/ml.^48,49^ All models were adjusted following the same procedure outlined previously, resulting in models with dichotomic CSF Aβ42 being adjusted by core covariates and log_10_(CSF p-tau), models with dichotomic CSF t-tau being adjusted by core covariates and log_10_(CSF Aβ42), and models with dichotomic CSF p-tau being adjusted by core covariates, physical activity, and log_10_(CSF Aβ42).

Linear mixed model analysis (LMM) was performed for longitudinal data, using the *lme* function in the lme package implemented in R v4.0.3. Levels of log(Aβ42), log(p-tau) and log(t-tau) were dependent variables; each sleep variable, age, sex, *APOE*-ɛ4 status and their interaction with time (operationalised as interval between the first and the last CSF sampling) were included as fixed effects; and patient identity as a random effect in all models. All models were adjusted by the previously mentioned covariate selection procedure so that analyses with CSF Aβ42 as outcome were adjusted by core covariates and log_10_(CSF p-tau), and models with CSF t-tau or p-tau as outcomes, were adjusted by core covariates and log_10_(CSF Aβ42). For example, the model specification for CSF Aβ42 levels as outcome and PSQI Total score as sleep variable was: log(Aβ42) ∼ PSQI Total score + age + sex + *APOE*-ɛ4 status + research site + log(p-tau) PSQI Total score*time + age*time + sex*time + *APOE*-ɛ4 status*time + (1|Participant). Statistical analyses were performed using the Stata 15 software (StataCorp. 2017. Stata Statistical Software: Release 15. College Station, TX: StataCorp LLC) and R statistical software (R Core Team 2014. R: A Language and Environment for Statistical Computing, version v4.0.3. Available at: http://www.r-project.org).

## Results

### Subjects characteristics

Demographic and clinical characteristics of the study population are shown in Table 1.

**Table 1:** Demographic, genetic data, CSF, cognitive and clinical data of the sample

Variable	Entire sample (N = 1168)	Subsample with longitudinal data (N = 332)	
	Mean (SD)/count (%)	Mean (SD)/count (%)	*P* ^a^
**Demographic**			
Age (years)	64.7 (7.1)	65.5 (6.4)	0.058
Female, *n* (%)	678 (58.1)	176 (51.6)	0.034
Education (years)	14.8 (3.5)	14.4 (3.8)	0.066
**Cognitive and clinical data**			
MMSE score	29.1 (1.0)	29.0 (1.0)	0.583
Depression (GDS total score)	4.4 (4.4)	4.4 (4.5)	0.92
Anxiety (STAI total score)	62.5 (15.0)	63.1 (14.7)	0.468
BMI (kg/m^2^)	26.3 (4.4)	26.5 (4.1)	0.368
Physical activity, *n* (%)			
Not at all	123 (10.6)	36 (10.6)	0.995
Few times/year	87 (7.5)	28 (8.3)	0.552
2–3/month	79 (6.8)	29 (8.6)	0.297
Once a week	197 (16.9)	44 (13.0)	0.034
2–3/week	473 (40.6)	133 (39.3)	0.66
Daily	204 (17.5)	68 (20.1)	0.208
**Genetic and CSF biomarkers data**			
*APOE*-e4 carriers, *n* (%)	424 (36.8)	134 (39.8)	0.278
CSF Aβ42 (pg/mL)	1452.7 (708.9)	1338.7 (617.1)	0.008
CSF p-tau (pg/mL)	17.8 (8.6)	18.5 (9.5)	0.228
CSF t-tau (pg/mL)	207.9 (83.6)	213.6 (89.0)	0.279
Interval between CSF collection (years)	-	1.5 (0.5)	-

^a^ *P*-values from two-sample *t*-test (continuous variables) or two-sample test of proportions (categorical variables). GDS, Geriatric Depression Scale, STAI, State-Trait Anxiety Inventory, MMSE, Mini-mental State Examination, BMI, body mass index.

In summary, the mean age for the entire sample was 64.7 (SD = 7.1) and for the subsample with longitudinal data 65.5 (SD = 6.4). Among the full study population, 58.1% were female, whereas there was a slightly smaller percentage of females in the longitudinal analyses (51.6% female). Participants with longitudinal CSF data displayed significantly lower CSF Aβ42 levels (*P* = 0.008) compared with the entire sample. Table 2 reports sleep characteristics for the entire sample and for the subgroup with longitudinal data. In the whole study sample, 38.8% of individuals were characterised as poor sleepers based on the PSQI Total score cut off of > 5, compared with 37.4% of those with longitudinal data.

**Table 2:** Sleep quality characteristics at baseline

Variable	Entire sample (N = 1168)	Subsample with longitudinal data (N = 332)	*P* ^a^
	Mean (SD)/count (%)	Mean (SD)/count (%)	
Total PSQI score	5.2 (3.3)	5.0 (3.1)	0.286
Poor sleepers (Total PSQI > 5), *n* (%)	453 (38.8)	124 (37.4)	0.635
Sleep latency, *n* (%)			
≤ 15 min	445 (38.1)	138 (41.6)	0.253
16–30 min	478 (40.9)	134 (40.4)	0.854
31–60 min	176 (15.1)	41 (12.4)	0.214
>60 min	69 (5.9)	19 (5.7)	0.899
Sleep duration, *n* (%)			
>7 h	713 (61.0)	203 (61.1)	0.974
6–7 h	323 (27.7)	94 (28.3)	0.813
5–6 h	108 (9.3)	29 (8.7)	0.775
<5 h	24 (2.1)	6 (1.8)	0.776
Sleep efficiency, *n* (%)			
>85%	583 (49.9)	179 (53.6)	0.234
75–84%	326 (27.9)	90 (27.1)	0.773
65–74%	137 (11.7)	34 (10.2)	0.451
<65%	122 (10.5)	30 (9.0)	0.453
Sleep disturbance, *n* (%)			
0	89 (7.6)	19 (5.7)	0.238
1–9	844 (72.3)	261 (78.6)	0.020
10–18	229 (19.6)	51 (15.4)	0.080
19–27	6 (0.5)	1 (0.3)	0.616
Use of sleep medication, *n* (%)			
Not during past month	953 (81.6)	263 (79.2)	0.330
Less than once a week	67 (5.7)	27 (8.1)	0.112
Once or twice a week	41 (3.5)	7 (2.1)	0.200
Three or more times a week	107 (9.2)	35 (10.5)	0.448
Daytime dysfunction, *n* (%)			
0	661 (56.6)	185 (55.7)	0.778
1–2	449 (38.4)	132 (39.8)	0.664
3–4	54 (4.6)	13 (3.9)	0.582
5–6	4 (0.3)	2 (0.6)	0.508

^a^ *P*-values from a two-sample *t*-test (continuous variables) or two-sample test of proportions (categorical variables).

### Cross-sectional analyses

**Figure 2:**
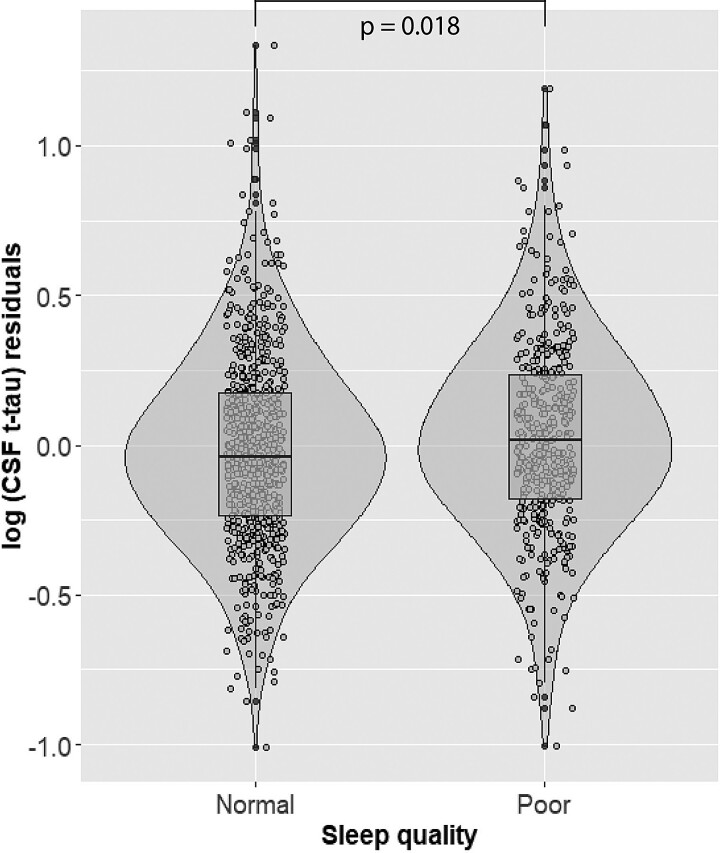
**Main effect of PSQI binary sleep category on CSF t-tau levels.** On the X-axis are represented participants’ groups categorized as normal sleep group (PSQI ≤ 5) or poor sleep group (PSQI >5). On the Y-axis are represented the residuals of log-transformed t-tau levels, after regressing out the effect of age, sex, site of data collection, *APOE*-ɛ4 carriership, body mass index, physical activity and CSF Aβ42 levels. Presented *P*-values are derived from multivariate linear regression analyses.

**Figure 3:**
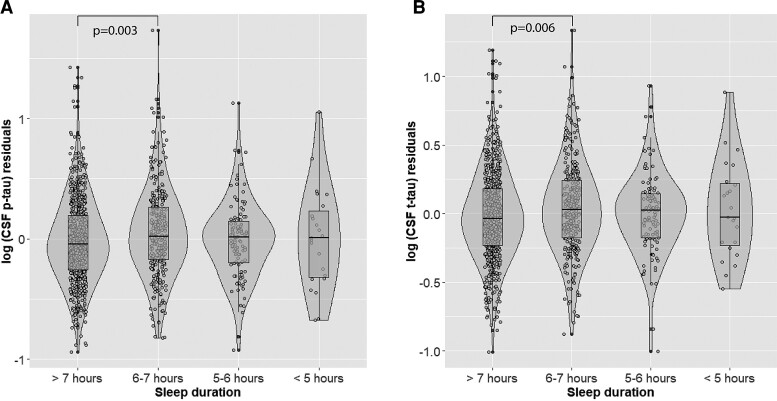
**Main effects of sleep duration on CSF p-tau and t-tau levels.** On the X-axis are represented participants’ groups categorised based on sleep duration >7 h, 6–7 h, 5–6 h and < 5 h of sleep. On the Y-axis are represented the residuals of log-transformed CSF p-tau (A) and t-tau (B) levels, after regressing out the effect of age, sex, site of data collection, *APOE*-ɛ4 carriership, body mass index, physical activity, and CSF Aβ42 levels. Presented p-values are derived from multivariate linear regression analyses.

**Figure 4:**
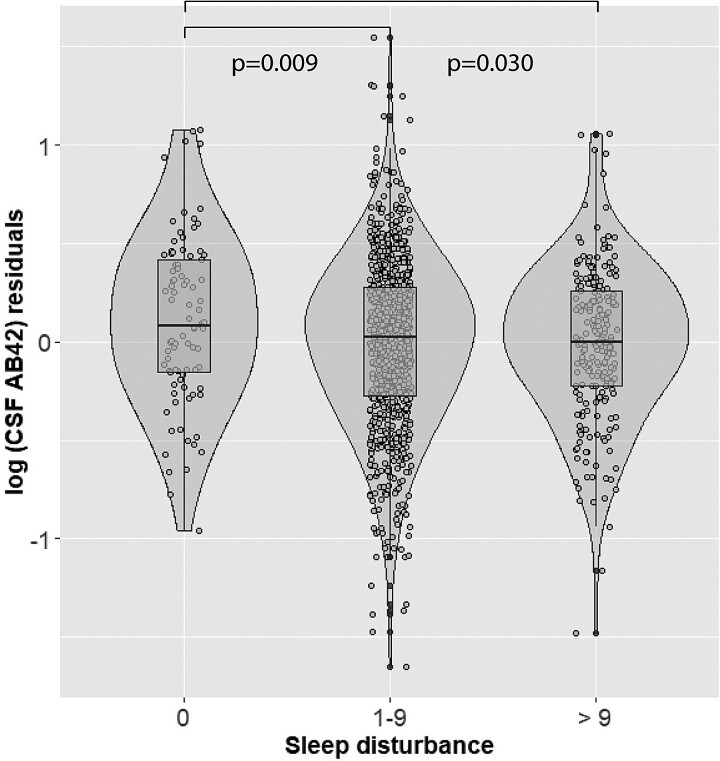
**Main effect of sleep disturbance on CSF Aβ42 levels.** On the X-axis are represented participants with sleep disturbance scores (PSQI component 5) of 0, 1–9 or >9. On the Y-axis are represented the residuals of log-transformed CSF Aβ42 levels, after regressing out the effect of age, sex, site, *APOE*-ε4 carriership, anxiety (State-Trait Anxiety Inventory), and CSF p-tau levels. Presented p-values are derived from multivariate linear regression analyses.

Poor sleep quality (PSQI total > 5) was significantly associated with higher log_10_(CSF t-tau) (hereinafter CSF t-tau) (β= 0.044, *P* = 0.018) (Table 3, Fig. 2). Participants who reported sleeping 6–7 h displayed higher CSF p-tau levels than those with >7 h of sleep (β = 0.054, *P* = 0.028) (Table 3, Fig. 3). Shorter sleep duration was also significantly associated with higher CSF p-tau after dichotomizing sleep duration to > 7 h versus < 7 h (β = 0.069, *P* = 0.003) and higher log_10_(CSF t-tau) (β = 0.057, *P* = 0.006). A higher degree of sleep disturbance (1–9 versus 0 and >9 versus 0) was associated with lower log_10_(CSF Aβ42) (hereinafter (CSF Aβ42) (β=−0.125, *P* = 0.009; β=−0.121, *P* = 0.030) (Table 3, Fig. 4). No significant associations between the remaining PSQI components or total score and CSF Alzheimer’s disease biomarkers were found (Table 3).

**Table 3:** Effect of PSQI measures on CSF biomarkers at baseline

Variables	log(CSF Aβ42)		log(CSF *P*-tau)		log(CSF t-tau)	
	β Coefficient (95% CI)	*P*-value	β Coefficient (95% CI)	*P*-value	β Coefficient (95% CI)	*P-*value
Total PSQI score	−0.003 (−0.012 0.006)	0.809	0.002 (−0.005 0.008)	0.609	0.003 (−0.003 0.008)	0.344
Dichotomized PSQI score (ref. Total PSQI ≤5)	−0.013 (−0.064 0.039)	0.63	0.039 (−0.002 0.081)	0.062	0.044 (0.007 0.08)	0.018
Sleep latency (ref. ≤ 15 min)						
16–30 min	−0.039 (−0.094 0.015)	0.153	0.007 (−0.038 0.053)	0.759	0.001 (−0.039 0.041)	0.946
31–60 min	0.033 (−0.041 0.107)	0.382	−0.007 (−0.068 0.054)	0.824	−0.008 (−0.062 0.046)	0.767
>60 min	0.058 (−0.05 0.165)	0.29	−0.02 (−0.108 0.069)	0.665	−0.014 (−0.092 0.064)	0.728
Sleep duration (ref. > 7 h)						
6–7 h	−0.034 (−0.089 0.022)	0.232	0.069 (0.023 0.115)	0.003	0.057 (0.016 0.097)	0.006
5–6 h	−0.04 (−0.125 0.045)	0.356	0.019 (−0.052 0.09)	0.597	0.036 (−0.026 0.098)	0.259
<5 h	0.097 (−0.076 0.27)	0.272	−0.01 (−0.151 0.131)	0.89	0.014 (−0.11 0.138)	0.824
Sleep efficiency (ref. > 85%)						
75–84%	−0.036 (−0.093 0.021)	0.212	0.015 (−0.033 0.062)	0.545	0.015 (−0.027 0.057)	0.481
65–74%	−0.039 (−0.118 0.04)	0.335	0.029 (−0.036 0.094)	0.385	0.024 (−0.034 0.081)	0.418
<65%	−0.002 (−0.086 0.082)	0.961	0.039 (−0.03 0.108)	0.267	0.046 (−0.015 0.107)	0.138
Sleep disturbance (ref. 0)^a^						
1–9	−0.125 (−0.219 −0.031)	0.009	0.016 (−0.063 0.094)	0.695	0.034 (−0.035 0.104)	0.334
>9	−0.121 (−0.23 -0.012)	0.03	0.016 (−0.074 0.107)	0.722	0.028 (−0.052 0.108)	0.49
Daytime dysfunction (ref. 0)^b^						
1–2	−0.037 (−0.09 0.016)	0.173	0.018 (−0.025 0.06)	0.411	0.014 (−0.023 0.052)	0.448
>2	−0.032 (−0.15 0.087)	0.6	−0.085 (−0.182 0.011)	0.084	−0.06 (−0.145 0.025)	0.165

ref.: Level of reference. ^a^Categories corresponding to scores of ‘10–18’ and ‘19–27’ have been collapsed due to <20 observations in one category. ^b^Categories corresponding to scores of ‘3–4’ and ‘5–6’ have been collapsed due to <20 observations in one category. All models with log(CSF Aβ42) as an outcome are adjusted by age, sex, site of data collection, *APOE*-ɛ4 carriership, anxiety (State-Trait Anxiety Inventory) and log(CSF p-tau) levels. All models with log(p-tau) and log(CSF t-tau) levels as outcomes are adjusted by age, sex, site of data collection, *APOE*-ɛ4 carriership, body mass index, physical activity and log(CSF Aβ42) levels.

**Table 4:** Effect of baseline PSQI measures on longitudinal change in CSF biomarkers

Variables	log(CSF Aβ42)		log(CSF p-tau)		log(CSF t-tau)	
	β Coefficient (95% CI)	*P*-value	β Coefficient (95% CI)	*P*-value	β Coefficient (95% CI)	*P*-value
Time × total PSQI score	0.000 (−0.00002 0.000004)	0.227	0.000 (−0.00001 0.00001)	0.703	0.000 (−0.00001 0.00001)	0.957
Time × dichotomized PSQI score (ref. Total PSQI ≤5)	0.000 (−0.0001 0.00003)	0.295	0.000 (−0.00004 0.0001)	0.534	0.000 (−0.00005 0.0001)	0.868
Time × sleep latency (ref. ≤ 15 min)^a^						
16–30 min	0.000 (−0.0001 0.00005)	0.471	0.000 (−0.00005 0.00007)	0.749	0.000 (−0.0001 0.00003)	0.291
>30 min	0.000 (−0.0001 0.0001)	0.727	0.000 (−0.00007 0.0001)	0.699	0.000 (−0.0001 0.00005)	0.473
Time × sleep duration (ref. > 7 h)^b^						
6–7 h	−0.0001 (−0.0001 0.00001)	0.09	0.000 (−0.0001 0.00003)	0.358	0.000 (−0.0001 0.0001)	0.838
<6 h	0.000 (−0.0001 0.0001)	0.57	0.000 (−0.0001 0.0001)	0.868	0.000 (−0.0001 0.0001)	0.902
Time × sleep efficiency (ref. > 85%)						
75–84%	0.000 (−0.0001 0.00003)	0.269	0.000 (−0.00004 0.0001)	0.435	0.000 (−0.0001 0.0001)	0.76
65–74%	0.000 (−0.0001 0.0001)	0.529	0.000 (−0.0001 0.0001)	0.828	0.000 (−0.0001 0.0001)	0.988
<65%	0.000 (−0.0001 0.0001)	0.687	0.000 (−0.0001 0.0001)	0.48	0.000 (−0.0001 0.0001)	0.808
Time × sleep disturbance (ref. ≤ 9)^c^						
1–9	−0.0002 (−0.0004−0.0001)	0.006	0.000 (−0.0001 0.0001)	0.771	0.000 (−0.0001 0.0001)	0.712
>9	−0.0002 (−0.0004−0.0001)	0.005	0.000 (−0.0001 0.0002)	0.527	0.000 (−0.0001 0.0002)	0.742
Time × daytime dysfunction (ref. 0)^d^						
≥1	−0.0001 (−0.00012 0.00001)	0.125	0.000 (−0.00005 0.0001)	0.888	0.000 (−0.0001 0.00005)	0.959

ref.: Level of reference. ^a^Categories corresponding to ‘31–60 min’ and ‘>60 min’ have been collapsed due to <20 observations in one category. ^b^Categories corresponding to ‘5–6 h’ and ‘<5 h’ have been collapsed due to <20 observations in two categories. ^c^Categories corresponding to scores ≤ 9 and > 9 have been collapsed due to <20 observations in two categories. ^d^Categories corresponding to scores of ‘1–2’, ‘3–4’ and ‘5–6’ have been collapsed due to <20 observations in two categories. All models are adjusted by age, sex, *APOE*-ɛ4 carriership (and their interactions with time) and site of data collection (fixed effects). Additionally, models with log(CSF Aβ42) as outcome are adjusted by log(CSF p-tau), and models with log(CSF t-tau) or log(CSF p-tau) as outcomes, are adjusted by log(CSF Aβ42). A random intercept for each CSF biomarker and change over time (slope) are included as random effects.

Results with dichotomised CSF biomarkers’ levels as outcomes, closely resemble those of cross-sectional using continuous measures of CSF biomarkers. Specifically, shorter sleep duration (6–7 h of sleep as compared with >7 h) was associated with an increased odds ratio of abnormal CSF *p*-tau (OR = 1.948, CI [1.226, 3.097], *P* = 0.005) and CSF t-tau levels (OR = 1.839, CI [1.169, 2.894], *P* = 0.008) (Supplementary Table 2). A higher frequency of sleep disturbance (1–9 versus 0 and >9 versus 0) was associated with increased odds ratio of abnormal CSF Aβ42 (OR = 1.821, CI [1.031, 3.217], *P* = 0.039; and OR = 2.142, CI [1.111, 4.130], *P* = 0.023) respectively (Supplementary Table 2). No significant associations between the remaining PSQI components or total score and dichotomised CSF Alzheimer’s disease biomarkers were found (Supplementary Table 2).

Results from the analyses stratified by amyloid status revealed that in the amyloid negative group (N = 839), poor sleep quality (PSQI total > 5) was significantly associated with higher CSF p-tau (β = 0.042, *P* = 0.029) and higher CSF t-tau [(β = 0.037, *P* = 0.031) (Supplementary Table 3)]. Participants who reported sleep latency between 16–30 min as compared to <15 min, demonstrated lower CSF Aβ42 levels (β = −0.049, *P* = 0.015). Shorter reported sleep duration of 6–7 h as compared with >7 h was significantly associated with lower CSF Aβ42 levels (β = −0.041, *P* = 0.046) and higher CSF *p*-tau levels (β = 0.048, *P* = 0.025). Increased daytime dysfunction of (1–2 versus none), was also associated with lower CSF Aβ42 (β = −0.051, *P* = 0.008), higher CSF *P*-tau (β =0.046, *P* = 0.019) and t-tau (β = 0.037, *P* = 0.034) (Supplementary Table 3). In contrast, in the amyloid positive group (N = 329), no significant associations between PSQI components or total score and CSF Alzheimer’s disease biomarkers were found (Supplementary Table 3).

### Longitudinal analyses

In all LMM analyses*, time* was a significant main effect, reflecting that CSF sampling interval was sufficient to capture changes in CSF biomarker levels. There was a significant interaction between sleep disturbances and time, with greater sleep disturbances at baseline (1–9 versus 0 and >9 versus 0) being associated with a decrease in CSF Aβ42 over time (β = −0.0002, *P* = 0.006; β=−0.002, *P* = 0.005). There were no other significant interactions between other sleep measures and time for CSF t-tau or p-tau levels (Table 4).

## Discussion

This study shows that, in cognitively unimpaired adults, self-reported indicators of poor sleep quality are associated with CSF signatures of Alzheimer’s disease (namely, decreased CSF Aβ42 and increased CSF t-tau and p-tau levels) at baseline. Longitudinally, increased sleep disturbance at baseline predicted a steeper decrease in CSF Aβ42, after an average follow-up of 1.5 years. Understanding longitudinal predictors of the Alzheimer’s disease CSF signature provides potential biomarkers for progression and specific targets for intervention.

### Self-reported sleep disturbances are associated with both lower baseline CSF Aβ42 and decreasing CSF Aβ42 over time

The sleep disturbance component of the PSQI was most robustly related to CSF Aβ42, both at cross-sectional and longitudinal levels. This component incorporates a range of factors united in their tendency to interrupt sleep, including snoring, nocturia and uncomfortable breathing. There area range of possible explanations for this finding. Firstly, those reporting increased sleep disturbances may reflect a cohort within the study more likely to have sleep-disordered breathing, itself associated with a pathological beta-amyloid profile.^38,50^ Alternatively it is possible that sleep interruptions may impede initiation and duration of slow-wave sleep^51^ distorting sleep-dependent amyloid production/clearance mechanisms,^52–56^ with such abnormalities contributing to or even driving this CSF profile.

Exploring the extent of sleep disturbance may hold future promise clinically as a marker of abnormal CSF Aβ42 given that the risk for this profile was approximately two-fold in participants reporting any sleep disturbances overnight. Given the multiple underlying causes for sleep disturbances, future work identifying exact underlying aetiologies most connected with this profile would help to shed further light on the mechanism.

When stratified by amyloid status, further self-reported sleep metrics were associated with a baseline reduced CSF Aβ42 but only within the CSF Aβ42 negative group. These include a mid-level increase in sleep latency (15–30mins), a mid-level reduction in sleep duration (6–7 hrs) and the presence of a mid-level degree of daytime dysfunction score (1–2). This finding points tentatively towards these sleep abnormalities as causative factors or that different sleep abnormalities may influence the CSF profile at different stages of the disease continuum. However, more severe scores within these categories were statistically non-significant, showing smaller or even reversed effect sizes. Although this could reflect reduced power to detect change within these categories populated by lower numbers of participants, overall we suggest these specific results be interpreted with caution.

### Self-reported measures of sleep abnormality are associated with higher baseline CSF t-tau and p-tau

Impaired sleep quality as defined by Total PSQI > 5 was associated with increased t-tau at baseline. Shorter sleep duration was also related to higher levels of CSF p-tau and t-tau biomarkers, demonstrating that, on average, even 1 hour of sleep loss is related to the accumulation of pathological tau proteins. Although this statistically significant association was observed in only one of the sleep duration groups (6–7 h), it remained in an analysis using dichotomic sleep duration (> 7 h versus < 7 h). While these associations were not present for other more severe categories, this could be explained by loss of power in the context of a smaller group membership.

This is in line with recent evidence showing that short sleep duration is associated with increased dementia risk.^57^ Like Aβ, ISF levels of tau also fluctuate diurnally,^33^ with studies supporting the hypothesis that this is driven by increased neuronal activity during wakefulness versus sleep.^58,59^ Lower sleep efficiency has been associated with higher CSF levels of tau in cognitively unimpaired adults.^25^ Additionally, evidence has shown a faster rate of tau increase to be present in patients with OSA as compared to controls.^38^ We hypothesise that those participants in our study with shorter sleep duration would be expected to have commensurate reduced time within a low neuronal/synaptic activity state, leading to a detectable increased tau CSF level. Indeed, shorter sleep duration (6–7 hrs) was associated with an approximately two-fold risk of abnormal CSF p-tau and t-tau, raising the possibility that this could be a marker of clinical interest.

### Self-reported measures of sleep abnormality are not associated with longitudinal change in CSF t-tau or p-tau

In contrast, no significant associations were found involving baseline sleep abnormalities and longitudinal change in CSF tau levels. Whilst the absence of a relationship is possible, there are several alternative explanations. Firstly, whilst the study time frame may be sufficient to capture longitudinal change in CSF Aβ, it may be of inadequate length for CSF tau, as tau changes may be more prominent in the later stages of the disease continuum, especially, since this cohort is comprised of cognitively unimpaired individuals at the inception of the pathological events’ cascade.^60^ Secondly, CSF tau has been shown to follow a non-linear pattern during the preclinical phase of Alzheimer’s disease and this could mask potential longitudinal associations with sleep quality.^61^ Thirdly, bidirectional causality between tau pathology and sleep abnormalities may be implicated. For example, cerebral tau deposition has been associated with increased total sleep time observed in cognitively unimpaired adults and patients with Alzheimer’s disease.^62,63^ Hence, whilst initial shorter total sleep duration may be cross-sectionally associated with higher CSF tau, its cerebral deposition could contribute to the opposite clinical effect, nullifying longitudinal relationships.

### Other research findings

The largest previous cross-sectional study, in a cohort of 736 cognitively unimpaired individuals, revealed associations of reduced Aβ42 and increased ratio of t-tau/Aβ42 and p-tau/Aβ42 ratio with both reduced and excessive total sleep time, daytime dysfunction and a later bedtime, but only in female or *APOE*-ε4 carrying participants.^29^ In agreement, we also found associations between shorter total sleep time and higher CSF p-tau, but not lower CSF Aβ42; findings which extended to our whole population. Decreased sleep efficiency and increased wake time after sleep onset, as measured by actigraph,^27^ have been associated with low CSF Aβ42, and future amyloid deposition has been associated with decreased sleep efficiency, as measured by polysomnography.^64^ Whilst no statistically significant relationship in terms of self-reported sleep efficiency was found here, it is reasonable to suppose that increased sleep disturbances overnight will adversely impact on overall sleep efficiency and as such these findings share similarities.

In summary, our strongest findings were in cross-sectional and longitudinal associations with sleep disturbance, which is in line with another study demonstrating the relationship between ‘Sleep problems’ according to the Medical Outcomes Study Sleep Scale (MOSSS) in cognitively unimpaired individuals and low CSF Aβ42 and raised p-tau/t-tau.^28^ This finding in a large cohort, suggests that sleep disturbances, alongside representing a candidate biomarker plausibly able to predict future amyloid accumulation easily and non-invasively, could offer a target for intervention.

### Strengths and limitations

This study has several strengths. Firstly, to our knowledge, it utilises the largest cross-sectional and longitudinal population to date to investigate the relationship between sleep and Alzheimer’s disease biomarkers, with study procedures coordinated and harmonised across multiple sites. Secondly, it is amongst a limited group of studies focussing on the preclinical stage of the disease using the CSF biomarkers to capture the underlying pathology.

Nonetheless, this study is not without weaknesses. The use of sleep monitoring devices (*e.g.* actigraphy or polysomnography), as opposed to self-reported questionnaires, would have enhanced objectivity and allowed for more sensitive detection of sleep abnormalities. Moreover, the categorical nature of the PSQI dataset available hinders hypotheses testing of potential non-linear associations between sleep quality and Alzheimer’s disease pathological indicators. For example, within this dataset, total sleep time was unavailable as a continuous variable precluding assessment of the effects of excessive sleep (sleep duration > 7 h is the longest category). Nevertheless, PSQI is a validated tool, widely used and relationships between self-reported measures and early Alzheimer’s disease change are of substantial clinical interest.

Other limitations relating to CSF sampling and biomarkers include the lack of CSF Aβ40, which prevented the use of the more sensitive Aβ42/40 ratio as a biomarker for Alzheimer’s disease pathology^65^ and the fact that, even though CSF was collected before noon, specific times were not provided preventing adjustment to approximate true peptide concentrations. This may be relevant, since CSF metabolism highly depends on circadian rhythm, with well demonstrated cyclic patterns of amyloid levels.^66,67^ Additionally, slight differences between the composition of cross-sectional and longitudinal samples were found. Specifically, CSF Aβ within the follow-up cohort was lower than in the group providing only baseline data. This may have been due to corresponding differences in age and *APOE-ε4* status which we do not believe adversely impacts on analysis or results interpretation.

We also must acknowledge the drawbacks associated with the external validity of our study. The EPAD study population is comparatively highly educated and this may influence CDR score and speed of diagnosis compared to the general population. This, in combination with selection bias universally common to cohort studies of this type, may compromise real-world applicability. However, overall PSQI score and the proportion of poor sleepers (PSQI) across the included population were in keeping with large community samples.^68,69^ In a similar vein, individuals concurrently utilizing sleep medications were included in the analysis. The sub-cohort taking sleep medications may well be the most significantly affected by sleep disturbance and as such, their exclusion was not felt to be appropriate. Models were adjusted to account for sleep medication use to minimize the potential confounding effect.

Finally, statistically, no correction for multiple comparisons was made and findings should be interpreted accordingly. However, the hypotheses and the primary data analytical approach were clearly determined prior to analysis and in this context correction increases the risk of Type 2 Error.

## Conclusion

This study demonstrates that self-reported sleep quality is associated with Alzheimer’s disease biomarkers in a cognitively unimpaired population. Baseline self-reported indicators of poor sleep quality were associated with lower Aβ42 and higher p-tau and t-tau CSF levels, and predicted CSF Aβ42 reduction over time.

Together, whilst warranting further investigation, these results support sleep impairment prior to cognitive symptom onset in Alzheimer’s disease and underline the importance of investigating the links between sleep and Alzheimer’s disease pathology. Effective treatments for sleep disorders and interventions for sleep quality exist and their early implementation may therefore potentially mitigate the progression of cognitive decline.

## Supplementary Material

fcac257_Supplementary_Data

## Data Availability

The dataset used for the present study can be found in an online repository (http://epad.org/erap/).

## Abbreviations

Aβ =: amyloid-β
BMI =: body mass index
EPAD-LCS =: The European Prevention of Alzheimer’s Dementia Longitudinal Cohort Study
GDS =: Geriatric Depression Scale
ISF =: interstitial fluid
MCI =: mild cognitive impairment
MMSE =: Mini-Mental State Examination
OSA =: obstructive sleep apnoea
p-tau =: phosphorylated tau
PSQI =: Pittsburgh Sleep Quality Index
t-tau =: total tau
STAI =: State-Trait Anxiety Inventory
