# Supplementary material for: Cross-sectional and longitudinal association of sleep and Alzheimer biomarkers in cognitively unimpaired adults
Source: Brain Commun. 2022 Nov 3;4(6):fcac257. doi: 10.1093/braincomms/fcac257 (PMC9630979; doi:10.1093/braincomms/fcac257)
Supplement: fcac257_Supplementary_Data [file fcac257_supplementary_data.docx]

**Supplementary material**

**Supplementary Table 1.** **EPAD Inclusion and Exclusion Criteria.**

|  |
| --- |
| **Inclusion Criteria** |
| Age > 50 years |
| Fulfils balancing committee criteria |
| Able to read and write with a minimum of 5 years of formal education |
| Willing to consider participation in a potential future pharmaceutical trial |
| Have a study partner or can identify someone willing in principle to be a study partner |
| **Exclusion Criteria** |
| Research participants who fulfil diagnostic criteria for any type of dementia at screening |
| CDR ≥ 1 at screening |
| Known carriers of a Presenilin (PSEN) PSEN1, PSEN2, or APP mutation associated with Autosomal Dominant AD or any other neurodegenerative disease |
| Presence of any neurological, psychiatric or medical conditions associated with a long-term risk of significant cognitive impairment or dementia including but not limited to pre-manifest Huntington’s Disease, multiple sclerosis, Parkinson’s Disease, Down syndrome, active alcohol/drug abuse; or major psychiatric disorders including current major depressive disorder, schizophrenia, schizoaffective or bipolar disorder. |
| Any cancer of history of cancer in the preceding 5 years (excluding cutaneous basal or squamous cell cancer resolved by excision and localized prostate cancer in male subjects) |
| Any current medical conditions that are clinically significant and might make the subject’s participation in an investigational trial unsafe e.g. uncontrolled or unstable disease of any major organ system; history within the last 6 months of any acute illness of a major organ system requiring emergency care or hospitalisation, including re-vascularisation procedures; severe renal or hepatic failure; unstable or poorly controlled diabetes mellitus, hypertension, or heart failure; malignant neoplasms within the last 5 years; any clinically relevant abnormalities in blood parameters included in local TDC routine assessments; severe loss of vision, hearing or communicative ability; or any conditions preventing co-operation or completion of the required assessments in the trial as judged by the investigator. |
| Any contra-indications for MRI/positron emission tomography (PET) scan |
| Any contra-indications for Lumbar Puncture at visit 1 (including refusal of lumbar puncture procedure to collect sample) |
| Any evidence of intracranial pathology which, in the opinion of the Investigator, may affect cognition including but not limited to brain tumours (benign or malignant), aneurysm or arteriovenous malformations, territorial stroke (excluding smaller watershed strokes), recent haemorrhage (parenchymal or subdural), or obstructive hydrocephalus. |
| Participation in a clinical trial of an investigational product (CTIMP) in the last 30 days |
| Diminished decision-making capacity / not capable of consenting at Visit 1. |
| Unable to comply with protocol requirements in the opinion of the Investigator |
|  |

**Supplementary Table 2 Effect of PSQI measures on dichotomic CSF biomarkers status at baseline**

|  |  |  |  |  |  |  |
| --- | --- | --- | --- | --- | --- | --- |
|  | **Aβ +** | | **p-tau +** | | **t-tau +** | |
| **Variables** | Odds ratio (95% CI) | p value | Odds ratio (95% CI) | p value | Odds ratio (95% CI) | p value |
| Total PSQI score | 1.015 (0.971 - 1.062) | 0.503 | 1.039 (0.974 - 1.108) | 0.247 | 1.040 (0.978 - 1.106) | 0.208 |
| Dichotomized PSQI score (ref. Total PSQI ≤5) | 1.116 (0.827 - 1.506) | 0.473 | 1.364 (0.892 - 2.085) | 0.152 | 1.501 (0.990 - 2.274) | 0.056 |
| Sleep latency (ref. ≤15 minutes) |  | | | | | |
| 16-30 minutes | 1.045 (0.757 - 1.444) | 0.788 | 1.275 (0.784 - 2.073) | 0.328 | 1.294 (0.804 - 2.083) | 0.289 |
| 31-60 minutes | 0.860 (0.545 - 1.358) | 0.518 | 1.546 (0.829 - 2.884) | 0.171 | 1.579 (0.864 - 2.889) | 0.138 |
| >60 minutes | 0.860 (0.452 - 1.638) | 0.647 | 0.732 (0.237 - 2.254) | 0.586 | 0.645 (0.214 - 1.948) | 0.437 |
| Sleep duration (ref. >7 hours) |  | | | | | |
| 6-7 hours | 1.038 (0.746 - 1.446) | 0.824 | 1.948 (1.226 - 3.097) | 0.005 | 1.839 (1.169 - 2.894) | 0.008 |
| 5-6 hours | 1.318 (0.796 - 2.182) | 0.283 | 0.639 (0.249 - 1.639) | 0.352 | 0.754 (0.317 - 1.796) | 0.524 |
| <5 hours | 0.480 (0.129 - 1.791) | 0.275 | 2.088 (0.544 - 8.020) | 0.284 | 1.679 (0.452 - 6.237) | 0.439 |
| Sleep efficiency (ref. >85%) |  | | | | | |
| 75-84% | 1.253 (0.783 - 2.007) | 0.348 | 1.372 (0.703 - 2.678) | 0.354 | 1.371 (0.709 - 2.651) | 0.349 |
| 65-74% | 1.253 (0.783 - 2.007) | 0.226 | 1.372 (0.703 - 2.678) | 0.316 | 1.371 (0.709 - 2.651) | 0.315 |
| <65% | 0.985 (0.594 - 1.633) | 0.954 | 1.580 (0.812 - 3.075) | 0.178 | 1.872 (0.994 - 3.528) | 0.052 |
| Sleep disturbance (ref. 0)^a^ |  | | | | | |
| 1-9 | 1.821 (1.031 - 3.217) | 0.039 | 0.929 (0.379 - 2.275) | 0.872 | 1.271 (0.498 - 3.240) | 0.616 |
| >9 | 2.142 (1.111 - 4.130) | 0.023 | 1.157 (0.430 - 3.114) | 0.773 | 1.520 (0.550 - 4.206) | 0.42 |
| Daytime dysfunction (ref. 0)^b^ |  | | | | | |
| 1-2 | 1.141 (0.842 - 1.546) | 0.395 | 1.364 (0.882 - 2.110) | 0.162 | 1.102 (0.728 - 1.668) | 0.646 |
| >2 | 1.443 (0.729 - 2.859) | 0.293 | 0.454 (0.100 - 2.051) | 0.304 | 0.860 (0.291 - 2.540) | 0.784 |
|  |  |  |  |  |  |  |

ref.: Level of reference. ^a^Categories corresponding to scores of "10-18" and "19-27" have been collapsed due to <20 observations in one category. ^b^Categories corresponding to scores of "3-4" and "5-6" have been collapsed due to <20 observations in one category. All models with Aβ status as the outcome are adjusted by age, sex, site of data collection, APOE-ɛ4 carriership and log(CSF p-tau) levels. All models with p-tau status as the outcome are adjusted by age, sex, site of data collection, APOE-ɛ4 carriership, physical activity and log(CSF Aβ42) levels. All models with t-tau status as the outcome are adjusted by age, sex, site of data collection, APOE-ɛ4 carriership and log(CSF Aβ42) levels.

**Supplementary Table 3 Effect of PSQI measures on CSF biomarkers levels at baseline stratified by amyloid status**

|  |  |  |  |  |  |  |
| --- | --- | --- | --- | --- | --- | --- |
|  | **log(CSF Aβ42)** | | **log(CSF p-tau)** | | **log(CSF t-tau)** | |
| **Variables** | β Coefficient (95% CI) | p value | β Coefficient (95% CI) | p value | β Coefficient (95% CI) | p value |
| **Amyloid negative (n=839)** | | | | | | |
| Total PSQI score | -0.002 (-0.008 0.003) | 0.419 | 0.004 (-0.002 0.009) | 0.162 | 0.004 (-0.001 0.009) | 0.12 |
| Dichotomized PSQI score (ref. Total PSQI ≤5) | -0.019 (-0.056 0.019) | 0.328 | 0.042 (0.004 0.08) | 0.029 | 0.037 (0.003 0.07) | 0.031 |
| Sleep latency (ref. ≤15 minutes) |  |  |  |  |  |  |
| 16-30 minutes | -0.049 (-0.088 -0.01) | 0.015 | 0.035 (-0.007 0.077) | 0.102 | 0.025 (-0.013 0.062) | 0.194 |
| 31-60 minutes | -0.006 (-0.059 0.047) | 0.836 | 0.04 (-0.015 0.096) | 0.153 | 0.032 (-0.017 0.081) | 0.201 |
| >60 minutes | 0.047 (-0.029 0.124) | 0.224 | -0.03 (-0.11 0.05) | 0.462 | -0.024 (-0.095 0.047) | 0.506 |
| Sleep duration (ref. >7 hours) |  |  |  |  |  |  |
| 6-7 hours | -0.041 (-0.081 -0.001) | 0.046 | 0.048 (0.006 0.09) | 0.025 | 0.037 (0 0.074) | 0.052 |
| 5-6 hours | -0.003 (-0.066 0.06) | 0.927 | -0.006 (-0.072 0.061) | 0.864 | 0.011 (-0.048 0.07) | 0.723 |
| <5 hours | 0.04 (-0.075 0.156) | 0.494 | 0.009 (-0.11 0.127) | 0.886 | 0.011 (-0.094 0.117) | 0.832 |
| Sleep efficiency (ref. >85%) |  |  |  |  |  |  |
| 75-84% | 0.007 (-0.035 0.049) | 0.743 | 0.007 (-0.037 0.051) | 0.747 | 0.011 (-0.028 0.051) | 0.565 |
| 65-74% | -0.017 (-0.075 0.041) | 0.56 | 0.053 (-0.007 0.113) | 0.083 | 0.045 (-0.008 0.099) | 0.094 |
| <65% | -0.014 (-0.075 0.046) | 0.644 | 0.042 (-0.02 0.105) | 0.187 | 0.04 (-0.016 0.095) | 0.161 |
| Sleep disturbance (ref. 0)^a^ |  |  |  |  |  |  |
| 1-9 | -0.055 (-0.124 0.013) | 0.113 | 0.035 (-0.036 0.106) | 0.336 | 0.053 (-0.01 0.116) | 0.102 |
| >9 | -0.055 (-0.134 0.024) | 0.17 | 0.051 (-0.03 0.132) | 0.218 | 0.059 (-0.013 0.131) | 0.11 |
| Daytime dysfunction (ref. 0)^b^ |  |  |  |  |  |  |
| 1-2 | -0.051 (-0.089 -0.013) | 0.008 | 0.046 (0.008 0.085) | 0.019 | 0.037 (0.003 0.072) | 0.034 |
| >2 | -0.019 (-0.106 0.068) | 0.67 | -0.053 (-0.143 0.037) | 0.244 | -0.02 (-0.1 0.06) | 0.617 |
| Continues on the next page | | | | | | |
| Continuation from the previous page | | | | | | |
|  | **log(CSF Aβ42)** | | **log(CSF p-tau)** | | **log(CSF t-tau)** | |
| **Variables** | β Coefficient (95% CI) | p value | β Coefficient (95% CI) | p value | β Coefficient (95% CI) | p value |
| **Amyloid positive (n=329)** | | | | | | |
| Total PSQI score | 0.005 (-0.006 0.016) | 0.385 | -0.009 (-0.026 0.007) | 0.27 | -0.004 (-0.019 0.011) | 0.575 |
| Dichotomized PSQI score (ref. Total PSQI ≤5) | 0.027 (-0.042 0.096) | 0.441 | 0.014 (-0.089 0.118) | 0.786 | 0.047 (-0.045 0.139) | 0.318 |
| Sleep latency (ref. ≤15 minutes)^e^ |  | | | | | |
| 16-30 minutes | -0.007 (-0.077 0.063) | 0.842 | -0.03 (-0.141 0.081) | 0.599 | -0.035 (-0.133 0.064) | 0.491 |
| >30 minutes | -0.023 (-0.113 0.068) | 0.625 | -0.138 (-0.278 0.001) | 0.052 | -0.115 (-0.239 0.01) | 0.071 |
| Sleep duration (ref. 0)^c^ |  | | | | | |
| 6-7 hours | 0.017 (-0.056 0.091) | 0.641 | 0.116 (0 0.232) | 0.05 | 0.1 (-0.003 0.204) | 0.058 |
| <6 hours | -0.01 (-0.111 0.09) | 0.84 | -0.023 (-0.181 0.135) | 0.778 | 0.017 (-0.124 0.158) | 0.814 |
| Sleep efficiency (ref. >85%) |  | | | | | |
| 75-84% | -0.041 (-0.115 0.033) | 0.278 | -0.046 (-0.163 0.071) | 0.442 | -0.043 (-0.148 0.061) | 0.412 |
| 65-74% | -0.054 (-0.157 0.049) | 0.305 | -0.095 (-0.259 0.068) | 0.252 | -0.084 (-0.23 0.061) | 0.256 |
| <65% | 0.013 (-0.098 0.124) | 0.817 | -0.034 (-0.209 0.14) | 0.698 | 0.006 (-0.15 0.161) | 0.942 |
| Sleep disturbance (ref. 0)^a^ |  | | | | | |
| 1-9 | -0.049 (-0.172 0.074) | 0.435 | -0.048 (-0.247 0.151) | 0.636 | -0.032 (-0.209 0.145) | 0.719 |
| >9 | 0.005 (-0.14 0.151) | 0.942 | -0.085 (-0.317 0.147) | 0.471 | -0.071 (-0.278 0.136) | 0.499 |
| Daytime dysfunction (ref. 0)^d^ |  | | | | | |
| >1 | 0.051 (-0.018 0.12) | 0.147 | -0.006 (-0.111 0.098) | 0.904 | -0.001 (-0.094 0.092) | 0.98 |
|  |  |  |  |  |  |  |

ref.: Level of reference. ^a^Categories corresponding to scores of "10-18" and "19-27" have been collapsed due to <20 observations in one category. ^b^Categories corresponding to scores of "3-4" and "5-6" have been collapsed due to <20 observations in one category. ^c^Categories corresponding to scores of "5-6 hours" and "<5 hours" have been collapsed due to <20 observations in one category. ^d^Categories corresponding to scores of "1-2", "3-4", and "5-6" have been collapsed due to <20 observations in two categories. ^e^Categories corresponding to scores of "31-60 minutes" and ">60 minutes" have been collapsed due to <20 observations in one category. All models with log(CSF Aβ42) as an outcome are adjusted by age, sex, site of data collection, APOE-ɛ4 carriership, anxiety (State-Trait Anxiety Inventory), and log(CSF p-tau) levels. All models with log(CSF p-tau) and log(CSF t-tau) status as outcomes are adjusted by age, sex, site of data collection, APOE-ɛ4 carriership, body mass index, physical activity, and log(CSF Aβ42) levels.
